# The expression and function of RASAL2 in renal cell carcinoma angiogenesis

**DOI:** 10.1038/s41419-018-0898-x

**Published:** 2018-08-29

**Authors:** Ke Hui, Yangyang Yue, Shiqi Wu, Yanan Gu, Bing Guan, Xinyang Wang, Jer-Tsong Hsieh, Luke S. Chang, Dalin He, Kaijie Wu

**Affiliations:** 1 Department of Urology, First Affiliated Hospital of Xi’an Jiaotong University, Xi’an, 710061 P.R. China; 2 Department of Urology, University of Texas Southwestern Medical Center, Dallas, 75235 TX USA

## Abstract

Patients with renal cell carcinoma (RCC) often develop resistance to antivascular drugs and eventually succumb to disease. However, the underlying molecular mechanism remains poorly understood. In this study, we demonstrated that RASAL2, a RAS GTPase-activating protein, played a tumor-suppressive role in RCC by targeting tumor angiogenesis. Firstly, we showed that RASAL2 was frequently epigenetically silenced in RCC, and its loss was negatively correlated with overall survival of RCC patients. Furthermore, we discovered that RASAL2 could inhibit RCC angiogenesis in vitro and in vivo. Mechanistically, we identified that RASAL2 could activate GSK3β by reducing Ser9 phosphorylation and subsequently decrease the expression of c-FOS and vascular endothelial growth factor A (VEGFA). Interruption of the p-GSK3β/c-FOS pathway with the specific inhibitor or small interfering RNA could reverse the expression of VEGFA, which may provide a new insight to prevent RCC from resistance to antivascular therapy.

## Introduction

Renal cell carcinoma (RCC) is a common malignancy in human genitourinary system. There are an estimated 63,990 new cases and 14,400 deaths from kidney and renal pelvic cancer in United States in 2017^1^. RCC has several subtypes, and clear-cell RCC (ccRCC) accounts for almost 65−70% of all RCC^2^. Almost all familial ccRCC and over 60% of sporadic ccRCC harbor the suppressor gene von Hippel-Lindau (VHL)-inactivated mutation^3^. About 20−25% of newly diagnosed RCC patients have developed distant metastasis, which has a poor prognosis^4^. Angiogenesis is a crucial process for the progression and metastasis of RCC, and antivascular drugs now are widely used in patients with metastatic RCC^5^. Unfortunately, drug resistance has been reported in recent years^6,7^, and the underlying molecular mechanisms of the development and metastasis of RCC are still poorly understood.

RASAL2, a unique RAS GTPase-activating protein (RAS-GAP), has been reported as a tumor-suppressor gene, which inhibits tumor progression in different types of cancer, such as luminal B breast cancer, ovarian cancer, lung cancer, nasopharyngeal carcinoma, and colorectal cancer^8–12^. In our previous study, we have also reported that RASAL2 was downregulated and inhibited epithelial–mesenchymal transition (EMT) and stemness in bladder cancer^13^. In contrast, Feng reported that RASAL2 played an oncogenic role in triple-negative breast cancer^14^. As seen, the role of RASAL2 in cancer is still controversial, and the expression and function of RASAL2 in RCC are still completely unknown.

In this study, we were the first to demonstrate that RASAL2 was downregulated in RCC and its expression level was correlated with the DNA promoter methylation status. Also, we observed that RASAL2 could negatively modulate RCC angiogenesis in vitro and in vivo, in which p-GSK3/c-FOS/VEGFA signaling would play a critical role. Therefore, our findings provide a new insight into the mechanism of RCC angiogenesis, in which RASAL2 may be a potential therapeutic target for RCC treatment.

## Results

### RASAL2 was downregulated in human RCC specimens and associated with RCC progression

**Fig. 1:**
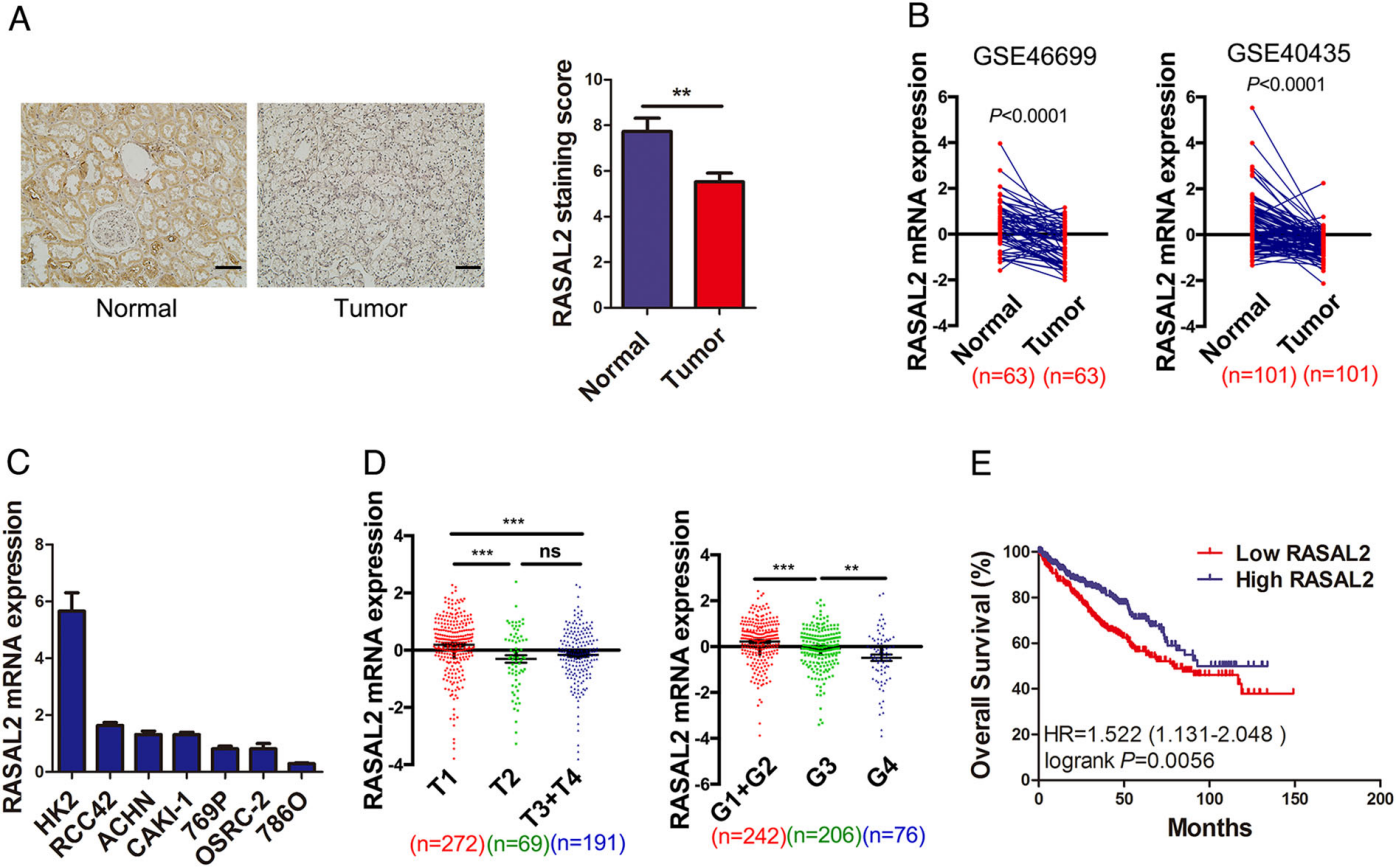
Expression of RSAL2 in RCC tissues and cell lines. **a** Immunohistochemistry staining of RASAL2 in normal kidney tissues (*n* = 18) and RCC tissues (*n* = 47). The scale bar is 40 µm. **b** RASAL2 mRNA expression in normal kidney tissues and matched RCC tissues from GEO database (GSE46699 and GSE40435). **c** Real-time quantitative PCR of RASAL2 mRNA expression in human RCC cell lines. **d** RASAL2 mRNA expression in human RCC tissues with different stages (T) and grades (g) from TCGA. **e** Kaplan–Meier analysis (long-rank test) of overall survival of RCC patients with high (*n* = 266) or low (*n* = 266) RASAL2 mRNA expression from TCGA. ^**^P < 0.01, ^***^P < 0.001, ns no significant

To investigate the expression pattern of RASAL2 in RCC tissues, we performed immunohistochemistry (IHC) staining in RCC and normal kidney specimens. As is shown in Fig. 1a, lower expression of RASAL2 protein was detected in RCC tissues compared to normal kidney tissues. Similarly, after analysis of RASAL2 mRNA expression in the paired RCC and normal kidney tissues from GEO databases (GSE46699 and GSE40435), we found the reduction of RASAL2 mRNA expression in RCC tissues compared to the paired normal kidney tissues (Fig. 1b). Moreover, as screened in Fig. 1c by real-time quantitative PCR assay, RASAL2 was highly expressed in HK2, an immortalized kidney tubular epithelial cell line, whereas a relatively lower expression was detected in all RCC cell lines. To be more important, after analyzing RASAL2 level in patients from TCGA cohort, we found that RASAL2 was inversely correlated with tumor stages and grades (Fig. 1d). In addition, we also found that RASAL2 expression was closely correlated with the overall survival of RCC patients (Fig. 1e).

Since RASAL2 DNA promoter was reported to be methylated in luminal B breast cancer^8^, we also analyzed the RASAL2 methylation profile in RCC tissues. Using publicly available TCGA methylation 450 data, we found a negative correlation between RASAL2 methylation and RASAL2 mRNA (Figure S1A), and a higher RASAL2 methylation was detected in tumor tissues compared to adjacent normal tissues (Figure S1B, C). Moreover, RASAL2 hypermethylation was associated with higher stages and grades in RCC patients (Figure S1D). Consistently, patients with hypermethylated RASAL2 showed a worse overall survival rates than those with hypomethylated RASAL2 (Figure S1E). To validate the loss of RASAL2 due to DNA promoter hypermethylation in RCC, we treated the OSRC-2 cell line with the hypomethylating agent 5-Aza-2-deoxycytidine (5-Aza). Indeed, we found that RASAL2 mRNA significantly increased in OSRC-2 cells treated with 5-Aza (Figure S1F). These results suggested that DNA promoter methylation might contribute to the downregulation of RASAL2 in RCC.

### RASAL2 suppressed RCC angiogenesis in vitro

**Fig. 2:**
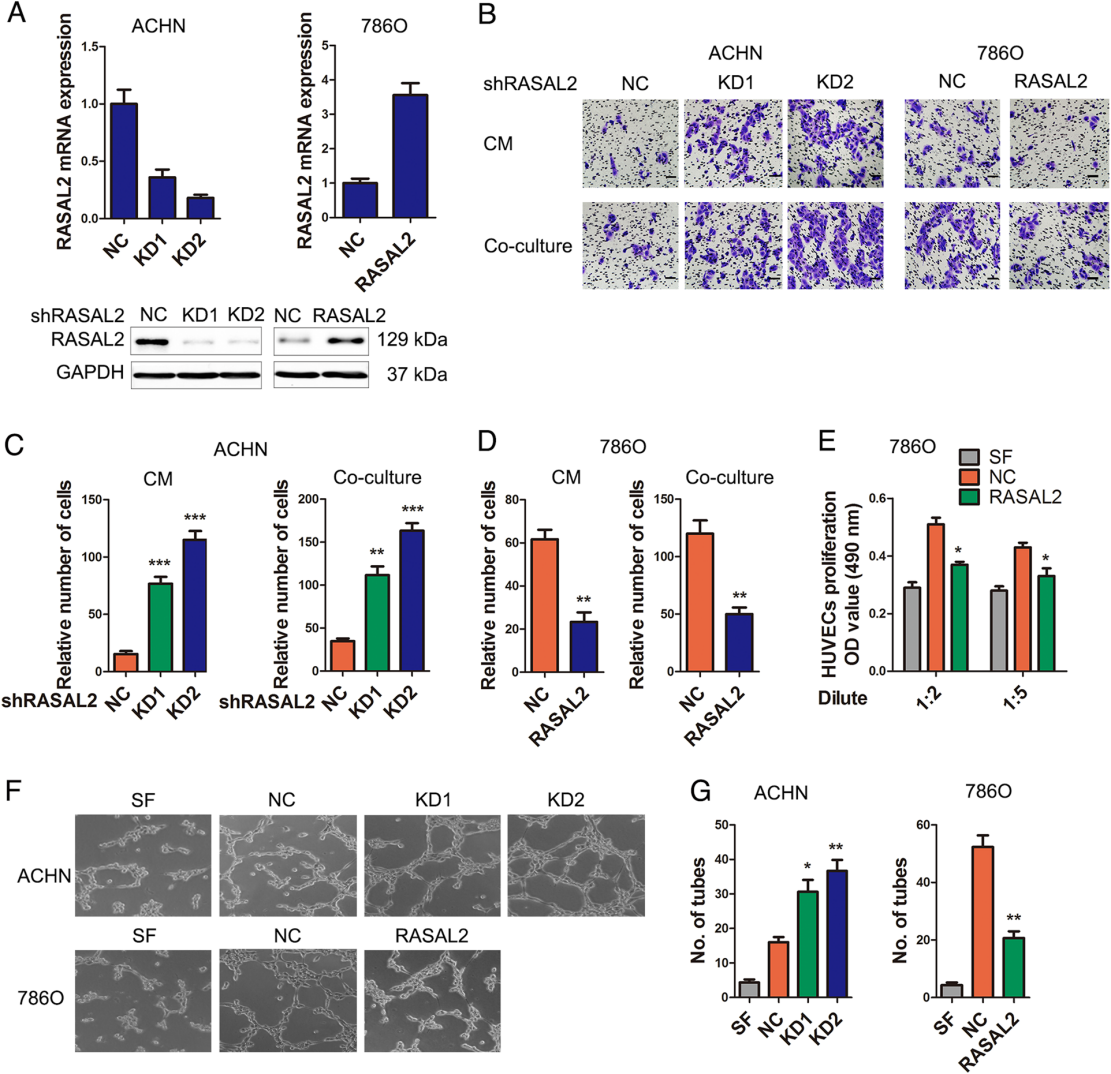
RASAL2 could modulate RCC angiogenesis. **a** Real-time quantitative PCR and western blot analysis of RASAL2 levels in ACHN cells transfected with RASAL2 shRNAs (KD) and negative control (NC), or 786O cells infected with RASAL2 lentivirus and negative control (NC). GAPDH was used as loading control. **b**, **c** Representative pictures and quantification analysis of the recruitment of HUVECs using the conditioned mediums (CMs) collected from ACHN sublines and cocultured system with ACHN sublines. The scale bar is 40 µm. **b, d** Representative pictures and quantification analysis of the recruitment of HUVECs using CMs collected from 786O sublines and cocultured system with 786O sublines. The scale bar is 40 µm. **e** MTT assay of HUVECs treated with SFM or diluted CMs from 786O sublines for 48 h. **f**, **g** Representative pictures and quantification analysis of HUVECs tube formation treated with serum free medium (SFM) or CMs from ACHN or 786O sublines. ^*^P < 0.05, ^**^P < 0.01, ^***^P < 0.001

To examine the effect of RASAL2 on RCC angiogenesis, we successfully established the stable ACHN sublines with endogenous RASAL2 knockdown (KD) and 786O sublines with ectopic RASAL2 overexpression (Fig. 2a). Indeed, we noticed that the conditioned mediums (CMs) and cocultured system from ACHN/KD cells enhanced the recruitment of human umbilical vein endothelial cells (HUVECs) (Fig. 2b, c), while the CMs and cocultured system from 786O/RASAL2 cells decreased the recruitment of HUVECs (Fig. 2b, d). Also, we found that the CMs from 786O/RASAL2 cells could inhibit cell proliferation of HUVECs (Fig. 2e). Moreover, the CMs from ACHN/KD cells promoted tube formation of HUVECs, whereas the CMs from 786O/RASAL2 cells suppressed tube formation of HUVECs (Fig. 2f, g). These data indicated the suppressor role of RASAL2 in RCC angiogenesis in vitro.

### RASAL2 inhibited VEGFA expression in RCC

**Fig. 3:**
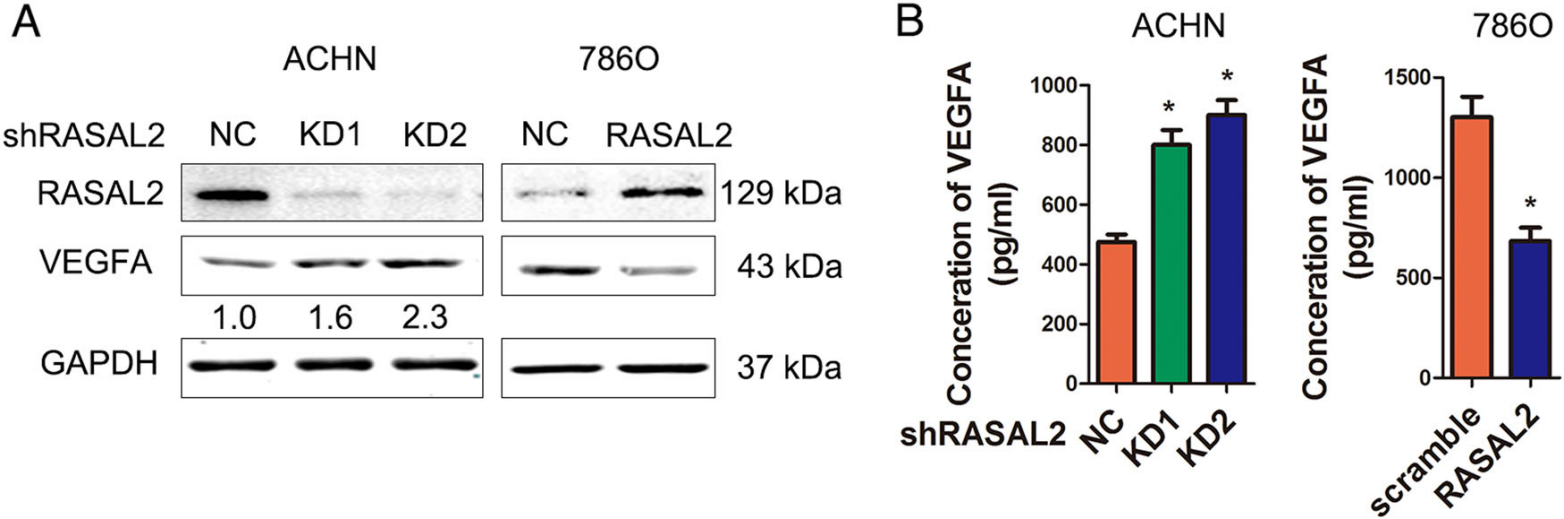
RASAL2 regulated the expression of VEGFA in RCC. **a** Western blot analysis of VEGFA expression in ACHN or 786O sublines. GAPDH was used as loading control, and the densitometric analysis of VEGFA expression was shown. **b** ELISA assay of VEGFA expression in CMs from ACHN or 786O sublines. ^*^P < 0.05

VEGFA was an important proangiogenic factor in different cancer^15^. Indeed, we found that VEGFA expression was upregulated in RASAL2-KD ACHN/KD sublines, but it was downregulated in RASAL2-overexpressed 786O sublines (Fig. 3a). Similar result was shown in OSRC-2 cells (Figure S2A). Moreover, VEGFA concentration was elevated in the CMs from ACHN/KD sublines but decreased in the CMs from 786O/RASAL2 sublines (Fig. 3b). These data indicated that RASAL2 inhibited RCC angiogenesis by reducing VEGFA expression.

### RASAL2 repressed RCC angiogenesis via p-GSK3β/c-FOS signaling pathway

Since VHL mutations occurred in 60–70% ccRCC cases and hypoxia-inducible factor (HIF) activated by VHL mutations could induce VEGFA expression for RCC angiogenesis^3,16^, we explored the association between RASAL2 and VHL status. Based on the analyses of VHL gene mutation data in RCC patients from TCGA database, we found a slight significant difference in terms of RASAL2 mRNA expression between VHL wild-type and mutation patients, whereas there was no significant difference in different mutation subtypes or mutation sublocations (Figs. S4B, and 4C, D). Moreover, HIF-1α level was detected with no difference between 786O/NC and 786O/RASAL2 sublines (Figure S4A). All these data failed to show the relationship between RASAL2 expression and VHL status in RCC.

**Fig. 4:**
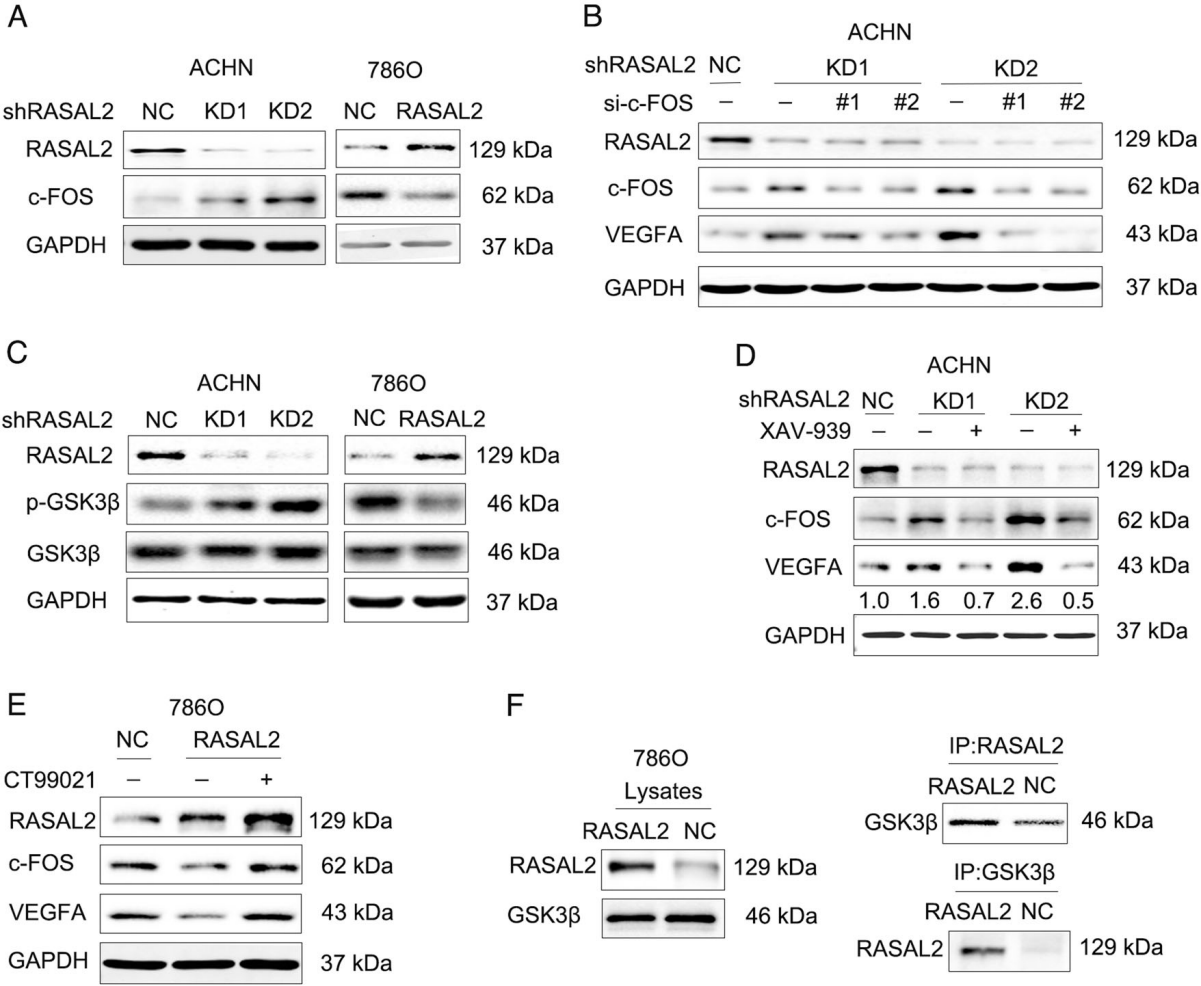
RASAL2 modulated VEGFA expression via p-GSK3β/c-FOS pathway in RCC. **a** Western blot analysis of c-FOS expression in ACHN or 786O sublines. **b** Western blot analysis of c-FOS and VEGFA expression in ACHN sublines transfected with c-FOS siRNA. **c** Western blot analysis of p-GSK3β^S9^ and GSK3β in ACHN or 786O sublines. **d**, **e** Western blot analysis of c-FOS and VEGFA in ACHN sublines treated with XAV-939 or 786O sublines treated with CT99021. The densitometric analysis of VEGFA expression was shown. **f** Immunoprecipitation assay of RASAL2 and GSK3β in 786O sublines. GAPDH was used as loading control

To further investigate the underlying mechanism of RCC angiogenesis, we referred to the KEGG pathway and found that c-FOS was involved in regulation of VEGF contributing to tumor angiogenesis. Indeed, we observed an increase of c-FOS in ACHN/KD sublines but decrease of c-FOS in 786O/RASAL2 or OSRC-2/RASAL2 sublines (Fig. 4a and Figure S2B). To further explore the role of c-FOS in RCC angiogenesis, we also applied siRNA strategy to KD c-FOS in ACHN/KD sublines and found that c-FOS KD could abolish the elevation of VEGFA after RASAL2 loss (Fig. 4b). We also observed a positive correlation between c-FOS and VEGFA in public available TCGA and GEO databases (Figure S3B).

Furthermore, we noticed that GSK3β phosphorylation on Ser9 significantly increased in ACHN/KD sublines but decreased in 786O/RASAL2 sublines (Fig. 4c), which indicated a regulation of GSK3β by RASAL2. Indeed, wnt pathway inhibitor XAV-939 could abolish the elevation of c-FOS and VEGFA after RASAL2-KD in ACHN cells (Fig. 4d). Consistently, the downregulation of c-FOS and VEGFA were rescued by GSK3β kinase inhibitor CT99021 in 786O/RASAL2 sublines or OSRC-2/RASAL2 sublines (Fig. 4e and Figure S2C). Moreover, there was a positive correlation between p-GSK3β^Ser9^ protein and VEGFA mRNA in RCC patients from TCGA cohort (Figure S3A). In addition, the Co-IP data indicated that RASAL2 could form a complex with GSK3β, which might contribute to the activation of GSK3β activity (Fig. 4f). There results suggested that RASAL2 suppressed RCC angiogenesis via p-GSK3β/c-FOS/VEGFA signaling pathway by interaction with GSK3β.

### RASAL2 inhibited RCC tumorigenecity and angiogenesis in vivo

**Fig. 5:**
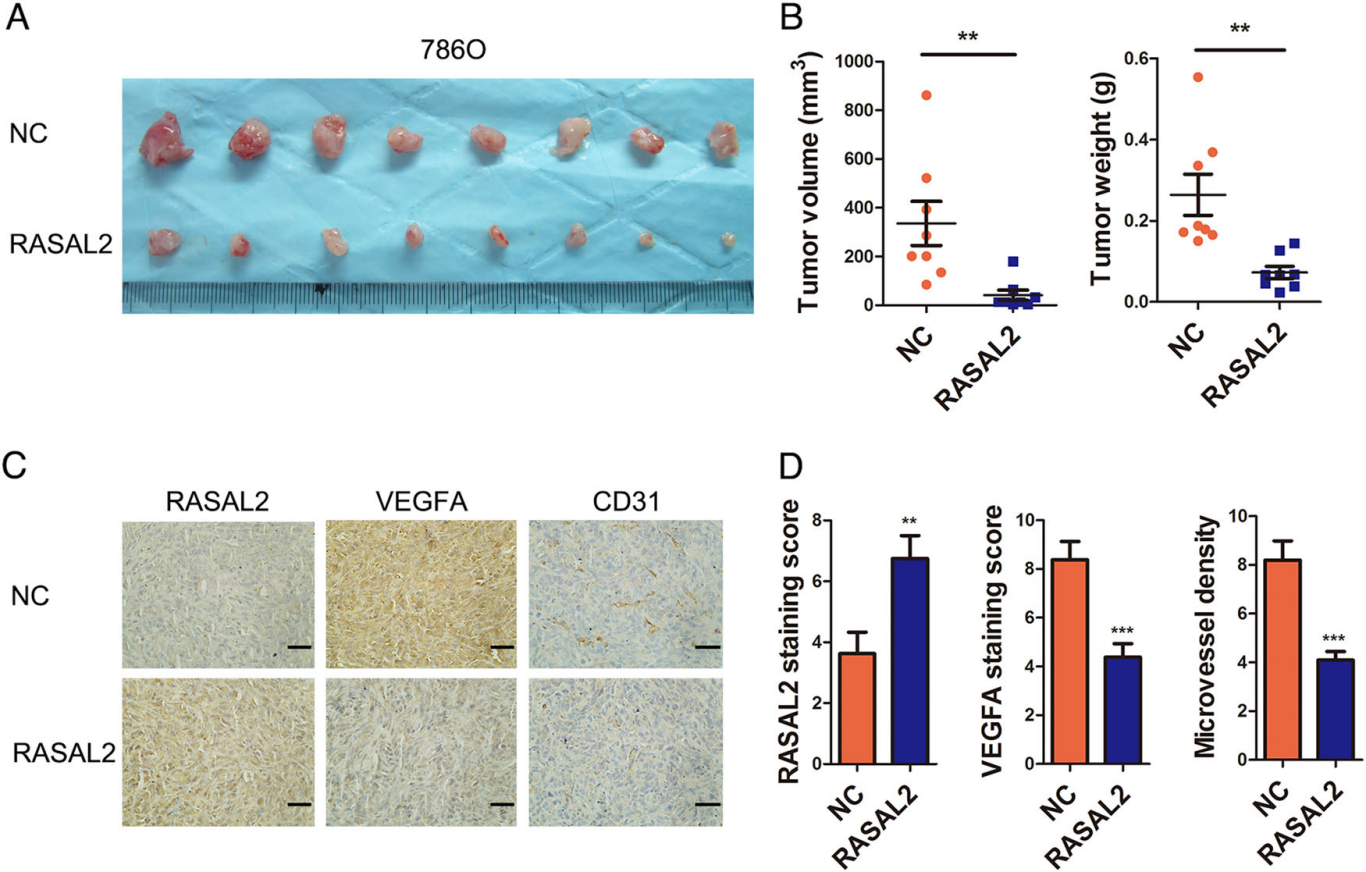
RASAL2 modulated RCC tumorigenicity and angiogenesis in vivo. **a**, **b** Photograph pictures and quantification analysis of subcutaneous xenografts in nude mice implanted with 786O/NC and 786O/RASAL2 sublines (*n* = 8). Xenograft weight (g) and volume (mm^3^) were measured. **c**, **d** Immunohistochemistry staining (IHC) of RASAL2, VEGFA and CD31 in xenograft tissues from 786O/NC or 786O/RASAL2 xenograft tumors. CD31 staining was used to evaluate the microvessel density (MVD) in xenografts. The scale bar is 40 µm

To further determine whether RASAL2 inhibited RCC tumorigenecity and angiogenesis in vivo, we established the subcutaneous xenograft using 786O sublines. We noticed that RASAL2 overexpression leaded to a reduced tumor weight and volume in comparison to control (Fig. 5a, b). Furthermore, we compared the expression of VEGFA and CD31 (vascular endothelial cell marker) in the xenograft tissues by IHC staining. In consistency with our in vitro findings, 786O/RASAL2 with higher RASAL2 expression presented a lower VEGFA staining and lower microvessel density (MCV) compared to control (Fig. 5c, d). These data implied that RASAL2 could negatively modulate RCC tumorigenecity and angiogenesis in vivo.

### RASAL2 was inversely correlated with VEGFA and MCV in human RCC specimens

**Fig. 6:**
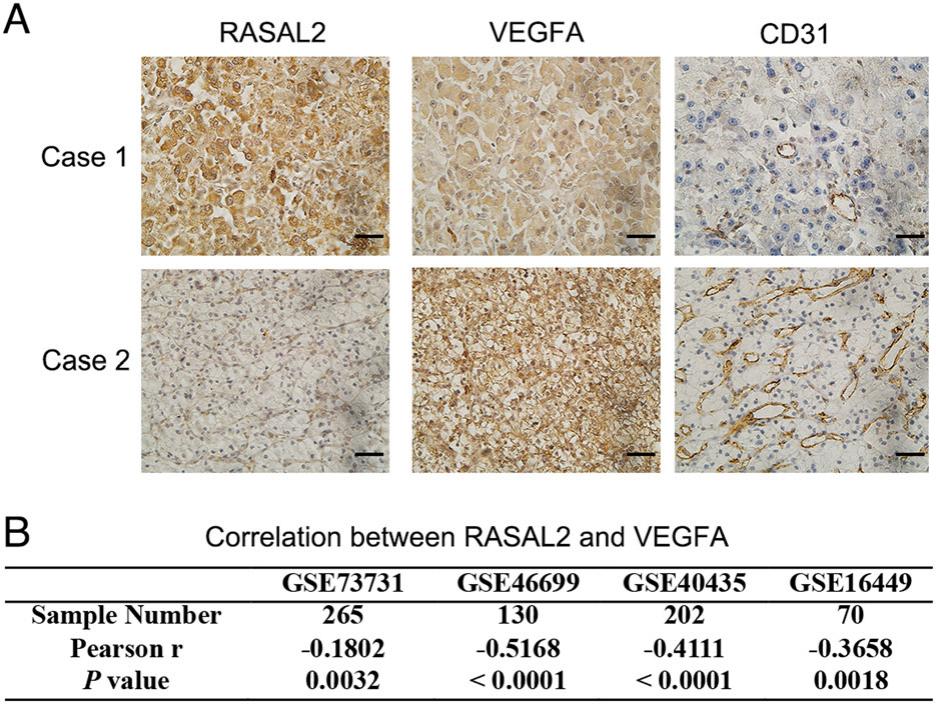
RASAL2 was associated with tumor angiogenesis in human RCC tissues. **a** Immunohistochemistry staining of RASAL2, VEGFA and CD31 in human RCC tissues. The scale bar is 40 µm. **b** Correlation between RASAL2 and VEGFA in RCC tissues from public available GEO databases (GSE73731, GSE46699, GSE40435, and GSE16449)

To strengthen the tumor-suppressor role of RASAL2 in RCC angiogenesis, we examine the association of RASAL2 and tumor angiogenesis in our clinical samples. Indeed, we found that there was a negative correlation between RASAL2 protein and VEGFA protein or MCV in RCC specimens (Fig. 6a). Also, using data from public available GEO databases, we observed an inverse correlation between RASAL2 mRNA and VEGFA mRNA (Fig. 6b). These data supported RASAL2 as a critical regulator in tumor angiogenesis of RCC.

## Discussion

Patients with aggressive or metastatic RCC always have a poor prognosis. Nowadays, although patients with metastatic RCC can initially benefit from the targeted therapy against angiogenesis, they will eventually acquire drug resistance^17,18^. Therefore, it is urgent and crucial to uncover the molecular mechanism of RCC progression or drug resistance to targeted therapy. Herein, we identify RASAL2 as a novel tumor suppressor in RCC, and it can inhibit RCC angiogenesis via p-GSK3β/c-FOS/VEGFA pathway.

RASAL2 is a RAS GTPase-activating protein (RAS-GAP), which can catalyze GTP into GDP and inactivate Ras^19^. McLaughlin was the first to report that RASAL2 was downregulated in luminal B breast cancer^8^. Subsequently, several studies reported that RASAL2 also decreased in various types of cancer, such as ovarian cancer, lung cancer, nasopharyngeal carcinoma, and colorectal cancer^9–12^. Indeed, we also previously found the downregulation of RASAL2 in bladder cancer^13^. However, other studies observed an upregulation of RASAL2 in triple-negative breast cancer and hepatocellular carcinoma^14,20^. In this study, we observed the downregulation of RASAL2 expression in RCC. Since RAS-GAPs were frequently inactivated by epigenetic mechanism^19,21^, we also detected the epigenetic status of RASAL2 and found a negative correlation between RASAL2 mRNA and DNA promotor methylation in RCC. In luminal B breast cancer and renal cell carcinoma, in which RASAL2 was downregulated, RASAL2 promoter methylation was enriched^8^. However, in hepatocellular carcinoma, RASAL2 promoter was hypomethylated with an elevated expression of RASAL2^22^. Consequently, one of the mechanisms for the different expression levels of RASAL2 in different tumor types was due to the methylation status of RASAL2 promoter. Similarly, DAB2IP, another member of the RAS-GAP family, was also downregulated due to DNA promotor hypermethylation in RCC^23^.

So far, the majority of research regarding RASAL2 was focused on EMT and invasion contributing to cancer metastasis. Herein, we found that besides EMT and invasion, the effect of RASAL2 on angiogenesis also contributed to RCC progression. By performing gain-of-function and loss-of-function studies in vitro and in vivo, we demonstrated that RASAL2 could suppress RCC angiogenesis by downregulation of VEGFA, which was critical for RCC progression and metastasis. We also observed that the effect of CM from 786O/RASAL2 cells on the recruitment, tube formation of HUVECs are more obvious than on HUVECs’ proliferation. Therefore, we supposed that besides VEGFA, other factors may be involved in the HUVECs’ proliferation. In ccRCC, there was another important molecular mechanism of angiogenesis, in which the inactivation of the *VHL* gene could result in the failure of HIF protein degradation and then induce VEGF expression^24^. In our study, we found that the role of RASAL2 in angiogenesis was independent of VHL status and HIF protein. Our findings suggest that besides VHL and HIF protein, RASAL2 may be another potential marker or target for RCC diagnosis and treatment.

DAB2IP was reported to form a complex with PP2A and GSK3β through its C2 domain, which further activated GSK3β in prostate cancer^25^. Herein, we found that RASAL2 interacted with GSK3β and then activated GSK3β through the reduction of Ser9 phosphorylation, and it ultimately reduced the expression of VEGFA in RCC, in which the nuclear oncogene c-FOS acted as an important bridge. Indeed, c-FOS played an important role in angiogenesis and promoted the VEGF expression in different disease^26–28^. However, the way in which RASAL2 regulates GSK3β in RCC needs further investigation since RASAL2 is not a phosphatase. Additionally, we need more efforts to uncover the relationship between GSK3β and c-FOS in RCC in our future work.

Taken together, the findings in our study revealed a novel mechanism of RCC angiogenesis contributing to progression and metastasis, in which loss of RASAL2 could facilitate RCC angiogenesis. Moreover, our results provide the evidence for understanding the critical role of p-GSK3β/c-FOS/VEGFA signaling in RCC development. As such, we have found that RASAL2 could be a potential prognostic marker or drug target for RCC diagnosis and treatment.

## Materials and methods

### Cell culture and reagents

Human RCC cell lines ACHN and 786O were purchased from the American Type Culture Collection. Human RCC cell line OSRC-2 cell line was obtained from National Platform of Experimental Cell Resources for Sci-Tech (Beijing, China), and human umbilical vein endothelial cell line HUVEC was kindly provided by Dr. Jer-Tsong Hsieh (University of Texas Southwestern Medical Center, Dallas, TX, USA). All cell lines were maintained in RPMI1640 medium for ACHN, 786 O and OSRC-2 cells or Dulbecco’s modified Eagle’s medium for HUVEC cells supplied with 10% fetal bovine serum (FBS) in a humidified incubator containing 5% CO_2_ at 37 °C. CT99021 and XAV-939 were purchased from Selleckchem (Houston, TX, USA). 5-Aza was purchased from Sigma-Aldrich (St Louis, MO, USA). The antibodies used were as follows: RASAL2 (Rabbit, GeneTex, Inc., Irvine, CA, USA), GAPDH (mouse, KangChen Bio-tech, Shanghai, China), VEGFA (Rabbit, abcam, Inc., Cambridge, Britain), c-FOS (Rabbit, Santa Cruz Biotechnology, Dallas, TX, USA), GSK3β (Rabbit, Cell Signaling Technology, Danvers, MA, USA), p-GSK3β^Ser9^ (Rabbit, Cell Signaling Technology, Danvers, MA, USA).

### Plasmid, siRNA transfection, and lentiviral infection

RASAL2 short hairpin RNA (shRNA) was used to stably KD RASAL2 in RCC cell lines. The sequence of RASAL2 shRNAs (RNAiCore, Academia Sinica, Taipei) were as follows: 5′-CCCTCGTGTTCTTGCTGATAT-3′ for KD1 and 5′- GCCTTCCACCTC TTCATAGTA-3′ for KD2. The small interfering RNA (siRNAs) were used to transiently KD c-FOS and the sequence of c-FOS siRNAs (GenePharma, Shanghai, China) were as follows: 5′-CAAGGUGGAACAGUUAUCUTT-3′ for KD1 and 5′-GACAGACCAACU AGAAGAUTT-3′ for KD2. Both shRNA and siRNA were transfected with X-tremeGENE HP DNA or X-tremeGENE siRNA transfection reagents (Roche Diagnostics, Indianapolis, IN, USA) according to the manufacturer’s protocol. RASAL2 overexpressing-lentivirus was purchased from GeneCopoeia (Guangzhou, China), and viral supernatant was used to infect the RCC cells in the presence of 8 µg/ml polybrene according to the manufacturer’s instructions.

### Real-time quantitative PCR

Total RNA was extracted from cells using a Total RNA Extraction kit from Fastagen (Shanghai, China), which was reverse-transcribed to cDNA using PrimeScript RT reagent kit (Takara, Dalian, China). Then the cDNA was studied by CFX96 real-time PCR system (Bio-Rad, Hercules, CA, USA) using SYBR-Green PCR Master Mix (Takara, Dalian, China). The sequence of gene-specific primers were as follows: RASAL2: F: 5′-AGCAGAAAGGTCCCCT CGTAG-3′; R: 5′-AGGGTGAGGTATTTGCAGTGT-3′; GAPDH, F: 5′-ATGGGGAAGGTGAAGGTCGG-3′; R: 5′-GACGGTGCCATGGAATTTG C-3′. GAPDH was used as loading control.

### CM collection and ELISA assay

RCC cells (5 × 10^5^) were seeded into 60 mm dish with 10% FBS medium. After 12 h, cells were washed with serum free medium (SFM), then cultured with 5 ml SFM for 24 h. The supernatant was collected and centrifuged to remove the debris. Enzyme-linked immunosorbent assay (ELISA) kit (RayBiotech Inc., Norcross, GA, USA) was used to determine the concentration of VEGFA in CM according to its manufacturer’s protocol.

### In vitro HUVECs recruitment assay and tube formation assay

The RCC cell-mediated HUVECs recruitment was evaluated by the Boyden chamber assay (Millipore, Bedford, MA, USA). Totally, 1 × 10^5^ cells/ml in 0.3 ml SFM were seeded into the upper chamber and 1 ml CM was added into the lower chamber. After 16-hour incubation, the upper surface of chambers were wiped with cotton swabs, then fixed in 4% paraformaldehyde and stained with crystal violet. Cell migration was measured by counting the number of cells attached to the lower surface of chambers. HUVECs were suspended with CM, and then added into 24-well plates coated with matrigel for tube formation. Pictures were taken after 4–6 h.

### Western blot assay

Western blot was performed as our previous study^13^. Cell lysates were prepared, and proteins were separated by 10–12% SDS-PAGE, and then transferred onto nitrocellulose membranes. After blocked with 5% nonfat milk, the membrane was incubated with primary antibodies at 4 °C overnight. After incubated with HRP secondary antibody at room temperature, the signal was detected using ECL detection system. GAPDH was used as loading control.

### Xenograft tumor model

Male BALB/c nude mice were subcutaneously injected with 2 × 10^6^ 786O sublines (NC and RASAL2) into both flanks. The mice were sacrificed and xenografts were harvested at 4 weeks, and then the tumor weight and volume was measured before subjected to IHC staining. The animal experiments were approved by the ethical committee of Xi’an Jiaotong University.

### Clinical specimens and IHC

To study the expression of RASAL2 in RCC tissues and its correlation with tumor angiogenesis, 47 ccRCC specimens and 18 normal kidney specimens were collected from the Department of Urology, The First Affiliated Hospital of Xi’an Jiaotong University. All specimens were used after written consent was obtained from patients. The IHC was performed as described in our previous study^13^.

### Bioinformatics and Statistical analysis

The public datasets (GSE73731, GSE46699, GSE40435, GSE16449, GSE15641, GSE53757, GSE19949, and GSE11151) were downloaded from NCBI GEO database (September 5, 2017). And RNA-sequencing-based mRNA expression data, HumanMethylation450 BeadChip array-based RASAL2 gene methylation data, the gene mutation data for VHL and the reverse phase protein array-based protein expression data for phosphorylated-GSK3β^S9^ were retrieved from cBioPortal for Cancer Genomics (August 1, 2017). All data were normalized to *z*-score before statistical analysis. All the statistical analyses were performed by GraphPad Prism version 6.0 software (GraphPad Software, CA, USA). All error bars in graphical data represent mean ± SEM and the difference between two groups were compared by the two-tailed Student’s *t* test. *P* < 0.05 was regarded as the threshold value for statistical significance.

## Electronic supplementary material

supplemental Fig.1

supplemental Fig.2

supplemental Fig.3

supplemental Fig.4

Supplemental Figure Caption
