# Supplementary figures and images for: The expression and function of RASAL2 in renal cell carcinoma angiogenesis
Source: Cell Death Dis. 2018 Aug 29;9(9):881. doi: 10.1038/s41419-018-0898-x (PMC6115459; doi:10.1038/s41419-018-0898-x)

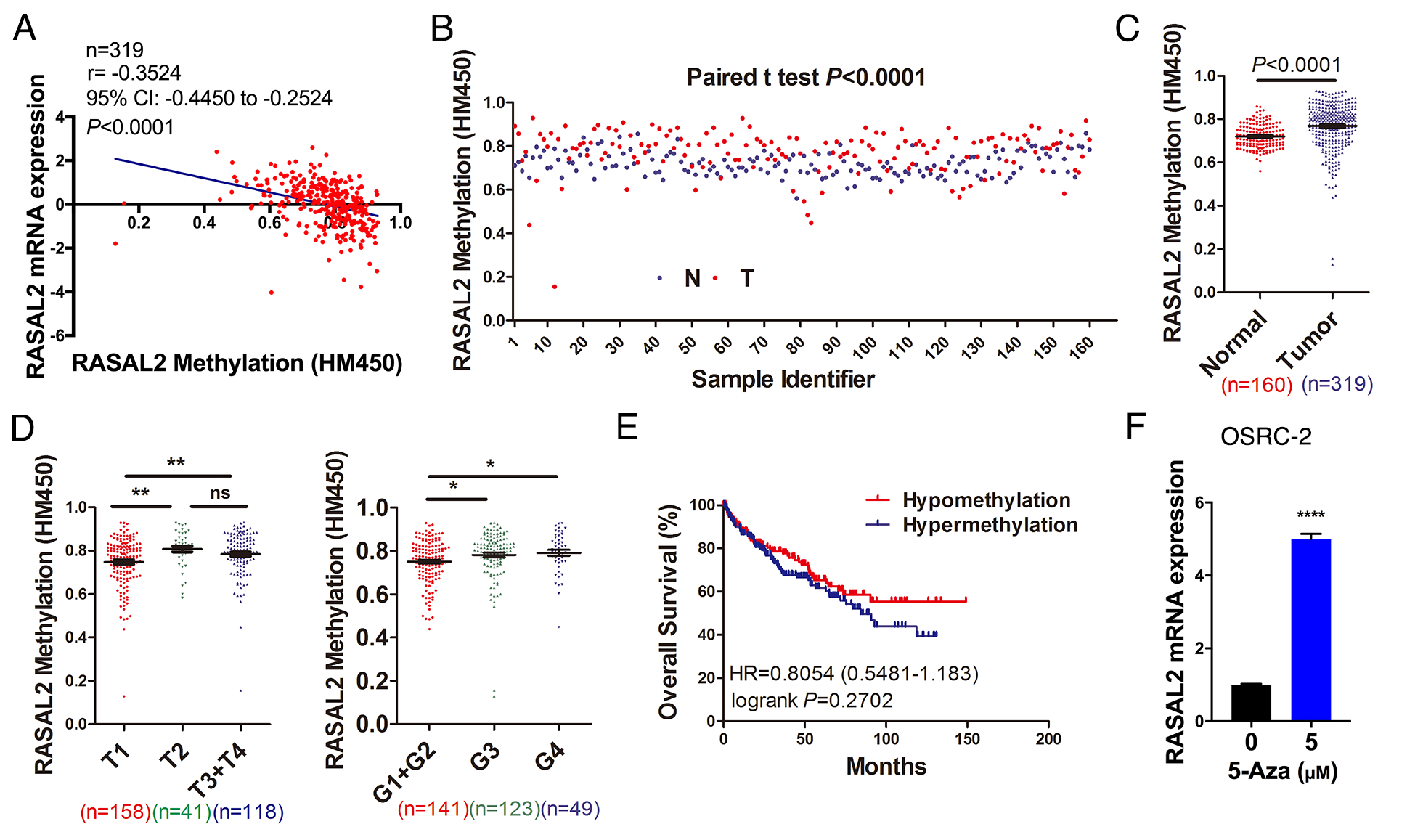

Supplement: Supplementary file 1 — supplemental Fig.1 [file 41419_2018_898_MOESM1_ESM.tif]

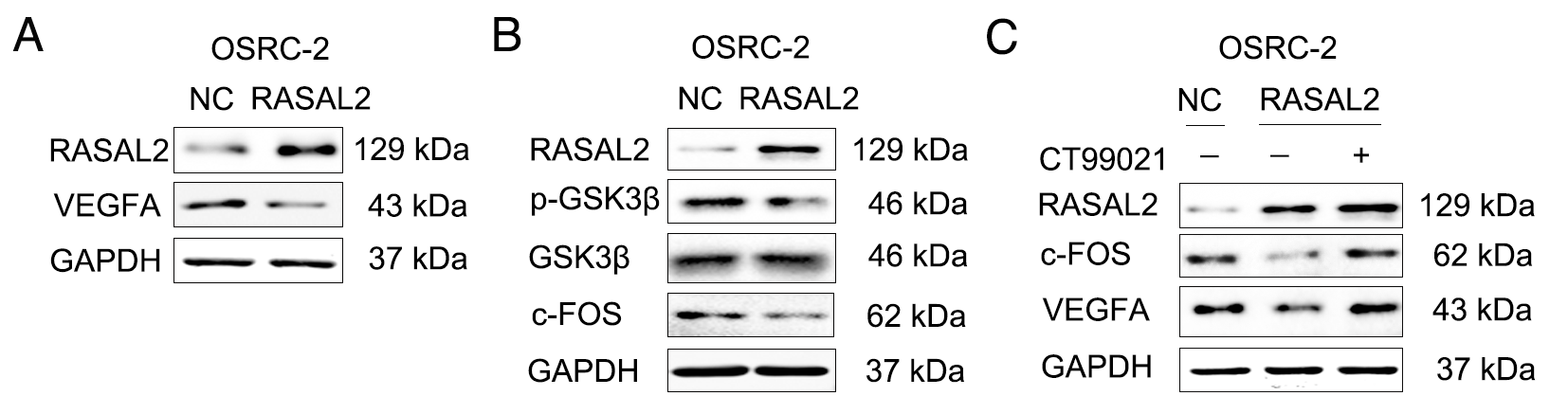

Supplement: Supplementary file 2 — supplemental Fig.2 [file 41419_2018_898_MOESM2_ESM.tif]

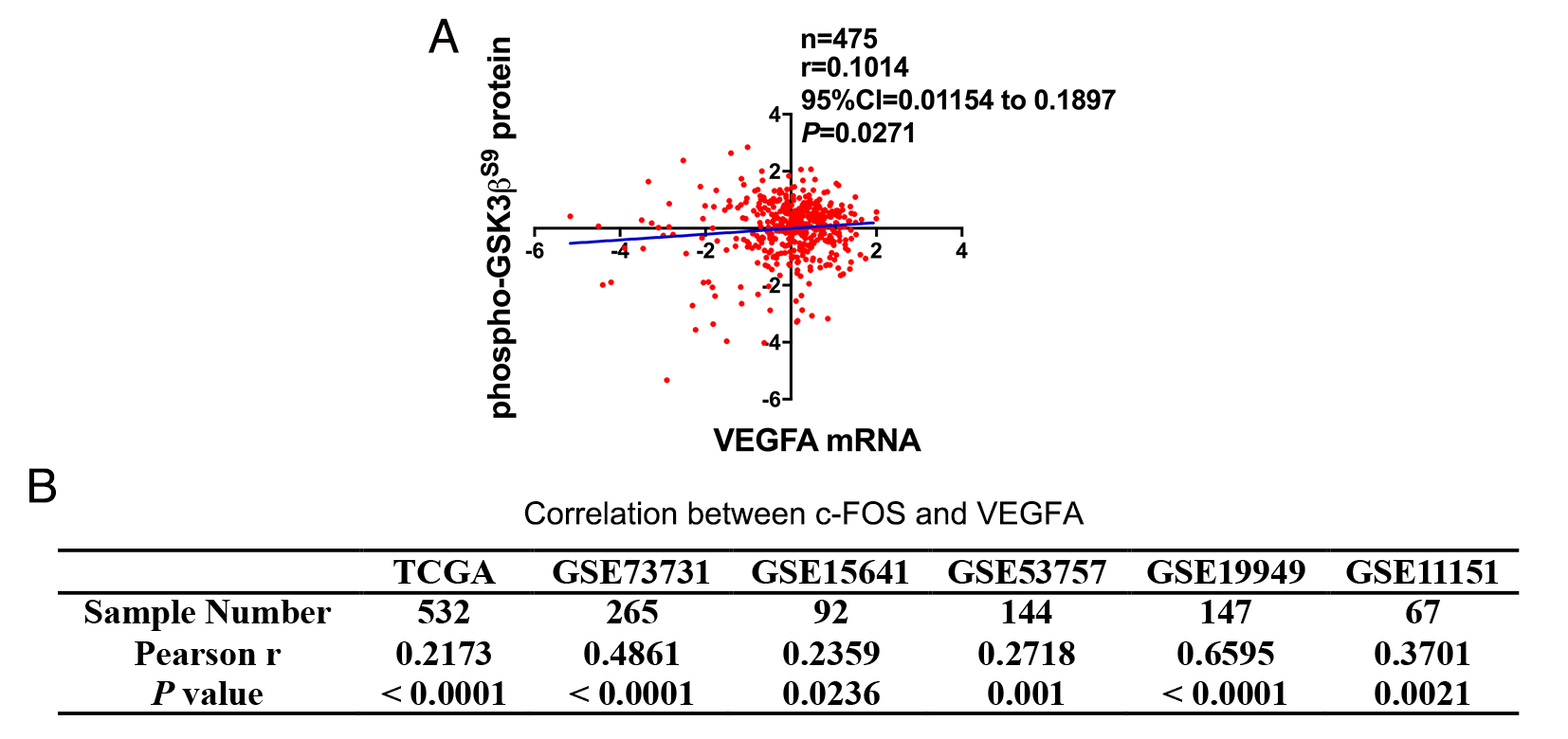

Supplement: Supplementary file 3 — supplemental Fig.3 [file 41419_2018_898_MOESM3_ESM.tif]

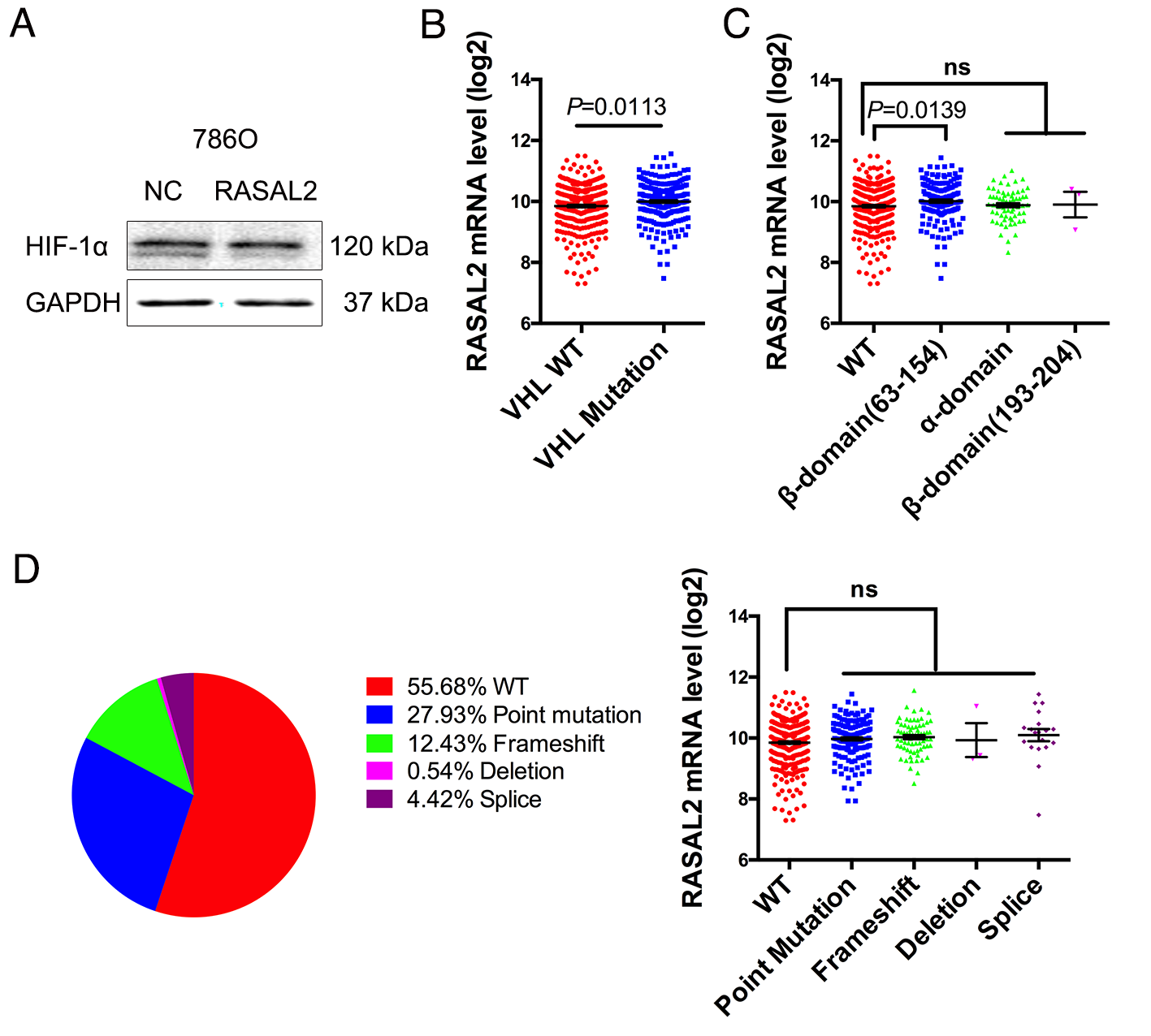

Supplement: Supplementary file 4 — supplemental Fig.4 [file 41419_2018_898_MOESM4_ESM.tif]
