# Supplementary material for: The expression and function of RASAL2 in renal cell carcinoma angiogenesis
Source: Cell Death Dis. 2018 Aug 29;9(9):881. doi: 10.1038/s41419-018-0898-x (PMC6115459; doi:10.1038/s41419-018-0898-x)
Supplement: Supplementary file 5 — Supplemental Figure Caption [file 41419_2018_898_MOESM5_ESM.docx]

**Supplemental Figure 1 The methylation status of RASAL2 in RCC**

A, The correlation between RASAL2 mRNA and RASAL2 DNA methylation levels in RCC patients from TCGA. B and C, RASAL2 DNA methylation levels in the paired RCC tissues (n=160) and normal kidney tissues (n=160) (B), or in the unpaired RCC tissues and normal kidney tissues (C) from TGCA. D, RASAL2 DNA methylation levels in RCC tissues with different stages (T) and grades (G) from TCGA. E, Kaplan-Meier analysis of overall survival of patients with low RASAL2 DNA methylation (n=157) and high RASAL2 DNA methylation (n=158) from TCGA. F, Real-time quantitative PCR analysis of RASAL2 in OSRC-2 cells treated with DMSO, 5-Aza (5μM) for 6 days. ^*^*P*<0.05, ^**^*P*<0.01, ^****^*P*<0.0001, ns=no significant.

**Supplemental Figure 2 RASAL2 regulated VEGFA expression via p-GSK3β pathway**

A and B, Western blot analysis of VEGFA, p-GSK3β^S9^, GSK3β and c-FOS in OSRC-2 infected with RASAL2 lentivirus and negative control (NC). C, Western blot analysis of c-FOS and VEGFA in OSRC-2/NC and OSRC-2/RASAL2 treated with CT99021.

**Supplemental Figure 3 The correlation between VEGFA and p-GSK3β/c-FOS pathway**

A, Correlation between VEGFA mRNA and p-GSK3β^S9^ protein in RCC tissues from TCGA. B, Correlation between c-FOS mRNA and VEGFA mRNA in RCC tissues from TCGA and public available GEO databases (GSE73731, GSE15641, GSE53757, GSE19949 and GSE11151).

**Supplemental Figure 4 The correlation between RASAL2 and VHL status in RCC**

A, Western blot analysis of RASAL2 and HIF-1α in 786O sublines. B, RASAL2 mRNA levels in VHL gene wild-type (WT) and mutated RCC patients from TCGA. C, RASAL2 mRNA in VHL WT and α-domain or β-domain mutated RCC patients from TCGA. D, RASAL2 mRNA in WT and different types of VHL-mutated RCC patients from TCGA. ns=no significant.
